# Genome-wide CRISPR screen reveals key role of sialic acids in PEDV and porcine coronavirus infections

**DOI:** 10.1128/mbio.01628-25

**Published:** 2025-08-06

**Authors:** Guanghao Guo, Mengjia Zhang, Zhuojia Xu, Peng Xi, Hongmei Zhu, Anouk Evers, Robert Jan Lebbink, Yifei Lang, Qigai He, Yao-Wei Huang, Tiehai Li, Berend Jan Bosch, Wentao Li

**Affiliations:** 1 National Key Laboratory of Agricultural Microbiology, Hubei Hongshan Laboratory, College of Veterinary Medicine, Huazhong Agricultural University 627716, Wuhan, China; 2 State Key Laboratory of Chemical Biology, Carbohydrate-Based Drug Research Center, Shanghai Institute of Materia Medica, Chinese Academy of Sciences 58298 , Shanghai, China; 3 Department of Medical Microbiology, University Medical Center Utrecht, Utrecht, the Netherlands; 4 College of Veterinary Medicine, Sichuan Agricultural University, Chengdu, China; 5 State Key Laboratory for Animal Disease Control and Prevention, South China Agricultural University 12526, Guangzhou, China; 6 Virology Section, Infectious Diseases and Immunology Division, Department of Biomolecular Health Sciences, Faculty of Veterinary Medicine, Utrecht University 8125, Utrecht, the Netherlands; Charite-Universitatsmedizin Berlin, Berlin, Germany

**Keywords:** PEDV, sialic acid, ST3GAL4, ST6GAL1, SLC35A1, coronaviruses

## Abstract

Porcine epidemic diarrhea virus (PEDV) is a globally distributed alphacoronavirus with economic importance that can cause severe watery diarrhea and even death in piglets. To identify host factors essential for PEDV infection, we performed a genome-wide CRISPR/Cas9 screen in human hepatocellular carcinoma cells (Huh7) using the highly virulent PEDV GIIb strain GDU. Several genes involved in the sialic acid and heparan sulfate biosynthesis pathway and cholesterol metabolism were highly enriched following PEDV selection. We validated that the host factor ST3 beta-galactoside alpha-2,3-sialyltransferase 4 (ST3GAL4), which catalyzes the transfer of sialic acid to sugar chains via α2,3-linked linkages, is important for PEDV infection. To systematically investigate the role of sialic acid in PEDV infection, we knocked out genes related to sialic acid synthesis. This led to a reduced abundance of sialic acid on the cell surface, which in turn inhibited PEDV adsorption and internalization. Furthermore, we found that both α2,3-linked and α2,6-linked sialic acids can serve as cellular attachment factors for PEDV. We conducted a glycan microarray screen to determine which sialoglycans are preferred by the PEDV spike protein. The results revealed that PEDV favors binding to α2,3-sialoglycans. Additionally, we found that not only current circulating PEDV strains but also other porcine coronaviruses rely on sialic acid for efficient infection. Collectively, our findings provide insights into critical host factors involved in PEDV infection and demonstrate that disrupting genes involved in sialic acid biosynthesis negatively affects the infectivity of multiple porcine enteric coronaviruses.

**IMPORTANCE:**

A wide range of viruses utilize sialic acid as receptors. Sialic acid binding may serve as a key determinant of viral host range. Different viruses exhibit distinct preferences for specific types of sialic acid linkages. However, it remains unclear which specific subtypes of sialic acid are utilized during PEDV infection. In this study, we performed CRISPR-based genome-wide knockout screening and identified ST3GAL4 as a key host factor for PEDV infection. Furthermore, we found that both α2,3-linked and α2,6-linked sialic acids can function as attachment factors for PEDV infection. A glycan microarray screen revealed that PEDV S1 shows the strongest binding preference for α2,3-linked and α2,8-linked sialosides. Sialic acids were also implicated in infections by other porcine enteric coronaviruses. Overall, our findings advance our understanding of viral entry mechanisms of PEDV and other swine coronaviruses and may provide avenues for designing antiviral strategies.

## INTRODUCTION

Porcine epidemic diarrhea (PED) is a highly contagious intestinal disease caused by PED virus (PEDV), which is characterized by vomiting, watery diarrhea, dehydration, and anorexia (1, 2). PEDV affects piglets more severely than it affects adult pigs and causes severe diarrhea in newborn piglets, with mortality rates ranging from 80% to 100%. First reported in the United Kingdom in 1971, PEDV was isolated in Belgium in 1977 and subsequently classified as a coronavirus (3). Initially, the low mortality rate of PEDV did not draw significant global attention. However, in 2013, the virus reached North America, rapidly spreading across the United States, wiping out over 10% of the hog population and causing severe damage to the local pork industry (4–6). In the following years, the virus spread widely around the world, initiating a second wave of PEDV outbreaks (7, 8). To date, PED has caused significant economic losses to the global pig industry, yet effective vaccines and treatments remain lacking.

PEDV belongs to the genus Alphacoronavirus within the subfamily Orthocoronavirinae of the family Coronaviridae. It is an enveloped, positive-sense, single-stranded RNA virus (3), with a genome length of approximately 28 kb, comprising seven open reading frames (ORFs). These encode 4 structural proteins (envelope, E; membrane, M; nucleocapsid, N; and spike, S), 16 nonstructural proteins (NSP1-16), and 1 accessory protein (ORF3) (9). Similar to other coronaviruses, the PEDV S protein mediates virus entry into cells. The S protein is a highly glycosylated type I transmembrane 180–200 kDa protein that consists of two subunits: the S1 subunit, responsible for receptor-binding, and the S2 subunit, which mediates membrane fusion. S1 comprises two main domains, the N-terminal domain (S1-NTD), and the C-terminal domain (S1-CTD), both of which could serve as receptor-binding domains (10).

Previous studies have shown that several host factors could serve as receptors for PEDV. For example, similar to TGEV, PEDV has been shown to use Aminopeptidase N (APN) as a receptor (11). However, recent studies have shown that the presence of APN is not essential for PEDV infection. In addition to APN, PEDV was shown to bind to sialic acid. PEDV pretreatment with trypsin or neuraminidase can induce hemagglutination of rabbit erythrocytes (12). Subsequent studies have shown that the PEDV S1 protein can bind to bovine or sheep mucins, and glycan array screening revealed that S1 binds to N-acetylneuraminic acid (Neu5Ac) (11). Similar to other coronaviruses, the sialic acid binding activity of PEDV was mapped to the N-terminal domain of S1 (13).

The synthesis of sialic acids is a complex process involving dozens of enzymes and intermediates (14). Neu5Ac is synthesized in the cytoplasm through a series of biochemical reactions and then transported to the nucleus. Neu5Ac is catalyzed by cytidine monophosphate n-acetylneuraminic acid synthase to form cytidine 5′-n-acetylneuraminic acid monophosphate (CMP-NeuNAc), an essential intermediate for the synthesis of sialic acid. CMP-NeuNAc is transferred by solute carrier family 35 member A1 (SLC35A1) to the Golgi, after which sialyltransferase transfers the sialic acid group to the sugar chain (15–17). Depending on how sialic acids are linked to penultimate sugar moieties, these sialytransferases are classified into four groups ST6Gal, ST6GalNAc, ST3Gal, and ST8Sia (14).

A wide range of viruses utilize sialic acid-containing molecules as receptors, including coronavirus, orthomyxoviruses, paramyxoviruses, and rotaviruses (18–22). The early stages of viral infection are triggered by virus binding to receptors on the surface of the host cell. Sialic acid binding may serve as a key determinant of viral host range. Different influenza strains exhibit distinct preferences for specific types of sialic acid linkages (α2-3 or α2-6). Avian influenza viruses predominantly interact with α2,3-linked sialic acid, while human influenza viruses bind more strongly to α2,6-linked sialic acid (23). The 1968 pandemic H3N2 influenza virus adapted to human-type receptor specificity through mutations in the hemagglutinin (HA) (23, 24). Preference specificity determines the site of infection, namely the intestinal tract of avian rich in α2,3-linked sialic acid and the upper respiratory tract of human rich in α2,6-linked sialic acid. However, it remains unclear which specific subtypes of sialic acid are utilized during PEDV infection.

CRISPR screening has emerged as a powerful tool to identify host factors required for viral infections, such as influenza A virus (IAV) (25, 26), porcine deltacoronavirus (PDCoV) (27), and porcine pseudorabies virus (28). PEDV propagated *in vitro* requires the addition of trypsin (-like) proteases in the culture medium. Trypsin (like) protease can damage normal cells, which can negatively affect the accuracy of CRISPR screening. Our previous study found that PEDV can be propagated in Huh7 cells without trypsin (-like) proteases (29), which facilitates the growth of signal cells. We performed CRISPR-based genome-wide knockout (KO) screening to identify host factors required for PEDV infection using a highly virulent strain. The ST3GAL4 gene encoding the sialyltransferase of α2,3-linked sialic acid was among the top hits of the screen. KO of ST3GAL4 inhibited PEDV infection, indicating that ST3GAL4 is essential for efficient infection by PEDV. Additionally, we found that PEDV can use α2,3-linked or α2,6-linked sialic acid as attachment factors and that removal of sialic acid inhibits the adsorption and internalization of PEDV. Our study further revealed that sialic acid enhances the entry of multiple coronaviruses into cells, suggesting that enzymes related to sialic acid biosynthesis could serve as targets for developing intervention strategies against porcine coronaviruses.

## RESULTS

### Genome-scale CRISPR screen identified host dependency factors for PEDV infection

**Fig 1:**
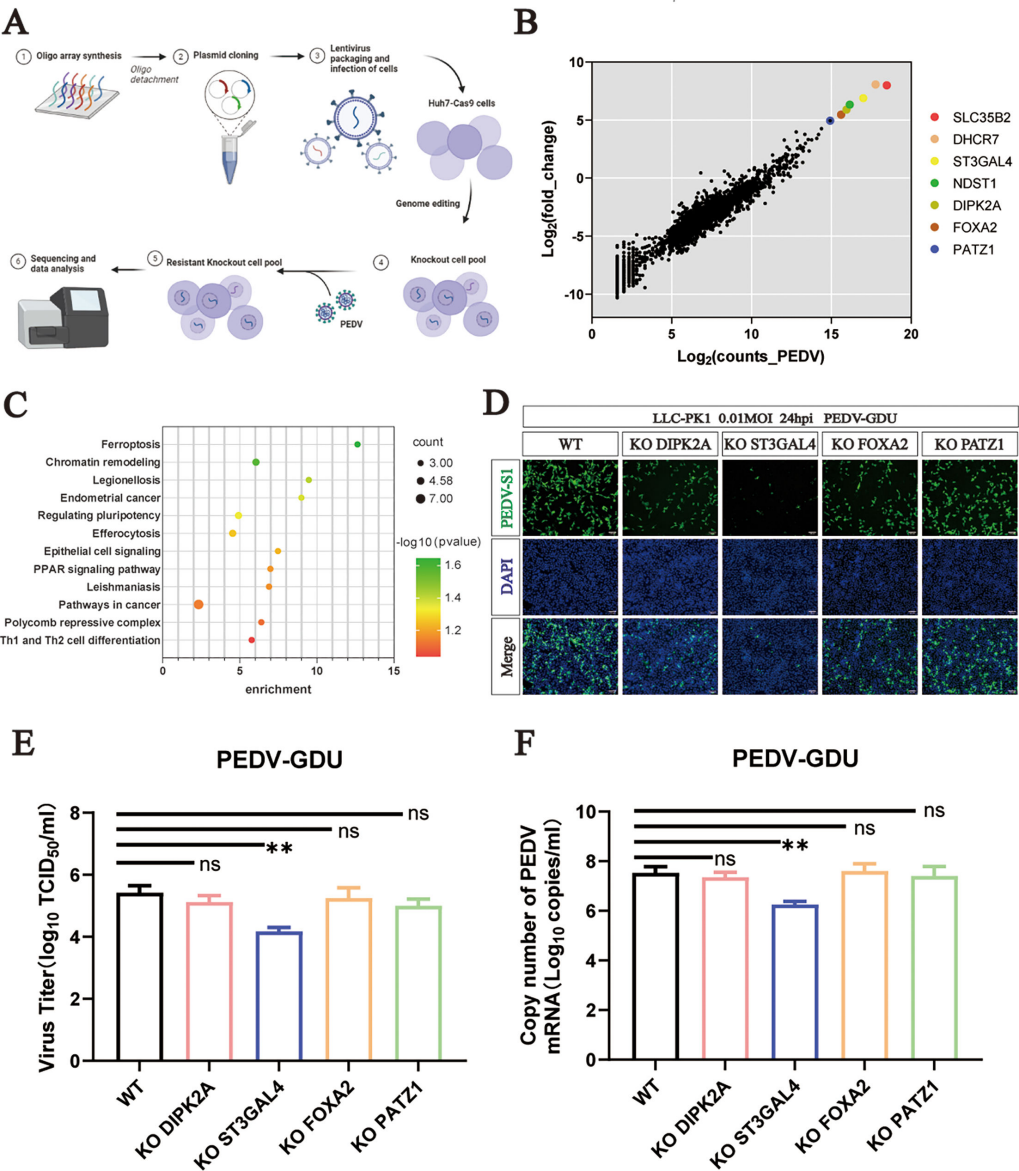
Genome-scale CRISPR screen identified host dependency factors for PEDV infection. (**A**) Overview of CRISPR screen in Huh7 cells. (**B**) Scatter plots reveal sgRNA-targeted sequence frequencies and the enrichment in control cells versus cells that survived PEDV infection. (**C**) KEGG pathway enrichment analysis for the top 50 sgRNA targets from the third PEDV challenge rounds. (**D**) PEDV infection of control and gene (DIPK2A, STGAL4, FOXA2, and PATZ1) KO LLC-PK1 cells. Cells were infected with PEDV (MOI = 0.1), and PEDV-infected cells were visualized by immunofluorescence using an S1-specific antibody. Scale bar = 100 µm. (**E**) TCID_50_ assay measuring PEDV titers in WT and KO LLC-PK1 cells. (**F**) qPCR quantification of PEDV N RNA in WT and KO LLC-PK1 cells. *P* values were determined by two-tailed unpaired *t*-tests. Error bars represent standard deviations from three independent experimental replicates. ns, no significant; *P < 0.05; **P < 0.01; ***P < 0.001; and ****P < 0.0001. Data are representative of at least three independent experiments.

To identify key host factors essential in PEDV replication, we screened a lentiviral-mediated genome-wide CRISPR screen in Cas9-expressing Huh7 cells that were mutagenized using a lentiviral gRNA library. For this screen, we performed three consecutive rounds of infection with a highly virulent PEDV (GDU strain, GIIb type) at an MOI of 0.1. Surviving cells were collected and single-guide RNA (sgRNA) sequences were PCR amplified and subjected to next-generation sequencing (NGS) (Fig. 1A). The top 10 ranked sgRNAs were identified by calculating the log2 fold change between control and surviving mutant cells. The top 10 most enriched genes after the third round of PEDV infection included SLC35B2, DHCR7, ST3GAL4, NDST1, DIPK2A, FOXA2, and PATZ1 (Fig. 1B). To identify the function of candidate PEDV-resistance genes, we conducted KEGG pathway enrichment analysis for top 50 ranked sgRNA targets. The analysis showed significant enrichment of genes related to sialic acid and heparan sulfate biosynthesis, as well as ferroptosis, following PEDV selection (Fig. 1C). Based on the results of our screen, we selected four genes for further validation, prioritizing them according to their rankings in the analysis. Porcine kidney proximal tubular epithelial cells (LLC-PK1) stably expressing Cas9 protein were used to construct KO cell lines. Polyclonal KO cells were then infected with PEDV at an MOI of 0.1 on day 9 post-sgRNA transduction. The results showed that KO of ST3GAL4 greatly inhibited PEDV infection (Fig. 1D). Moreover, we found that ST3GAL4 KO reduced both virus titers and viral RNA copies (Fig. 1E and F).

### ST3GAL4 is an important host factor in PEDV infection

**Fig 2:**
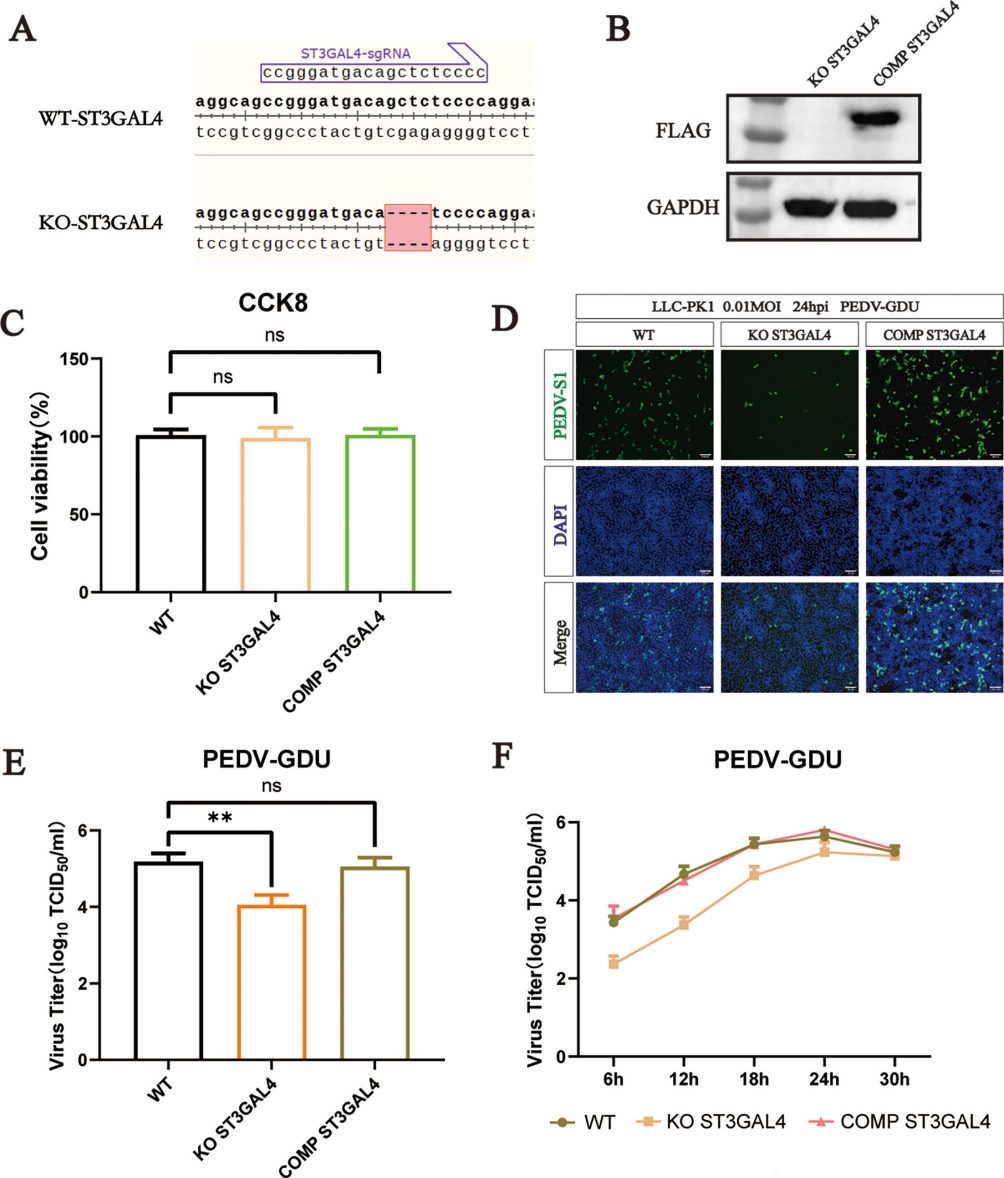
ST3GAL4 is an important host factor in PEDV infection. (**A**) Alignment of the nucleic acid sequences of WT and ST3GAL4 KO LLC-PK1 cells. (**B**) Western blot analysis to detect the expression of ST3GAL4-flag in ST3GAL4 KO LLC-PK1 and KO-ST3GAL4 complement LLC-PK1 cells. (**C**) Cell viability detection in WT, ST3GAL4 KO, and KO-ST3GAL4 complement LLC-PK1 cells by a CCK-8 kit. (**D**) Immunofluorescence assays for detection of the PEDV in WT, ST3GAL4 KO, and KO-ST3GAL4 complement LLC-PK1 cells following infection with PEDV (MOI = 0.1) at 24 hpi. Scale bar, 100 µm. (**E**) The viral titers of PEDV in WT, ST3GAL4 KO, and KO-ST3GAL4 complement LLC-PK1 cells infected with PEDV (MOI = 0.1) at 24 hpi were evaluated by TCID_50_ assay. (**F**) One-step growth curves of WT, ST3GAL4 KO, and KO-ST3GAL4 complement LLC-PK1 cells infected with PEDV (MOI = 0.1) measured by TCID_50_ assay. *P* values were determined by two-tailed unpaired *t*-tests. Error bars represent standard deviations from three independent experimental replicates. ns, no significant; *P < 0.05; **P < 0.01; ***P < 0.001; and ****P < 0.0001. Data are representative of at least three independent experiments.

Since ST3GAL4 KO cells exhibited the strongest inhibition of PEDV replication, we constructed a clonal ST3GAL4 KO LLC-PK1 cell line. A four-base pair deletion was identified at the sgRNA binding region compared to the wild-type (WT) sequence, verifying the KO of the ST3GAL4 gene in the cell line (Fig. 2A). To determine whether ST3GAL4 is required for PEDV replication, we constructed a ST3GAL4-KO-complement LLC-PK1 cell line, which stably expressed the ST3GAL4 gene (Fig. S1). Western blot analysis using a Flag-tag antibody confirmed stable expression of ST3GAL4 in the complemented cells (Fig. 2B). No significant difference was observed in cell viability among ST3GAL4 KO, complement, and WT cells (Fig. 2C). Knocking out ST3GAL4 significantly reduced virus titers after infection, while PEDV replication was fully restored upon ST3GAL4 complementation (Fig. 2D through F). These results demonstrate that ST3GAL4 is an important host factor for PEDV infection.

### PEDV utilizes both α2,6-linked and α2,3-linked sialic acid to infect cells

**Fig 3:**
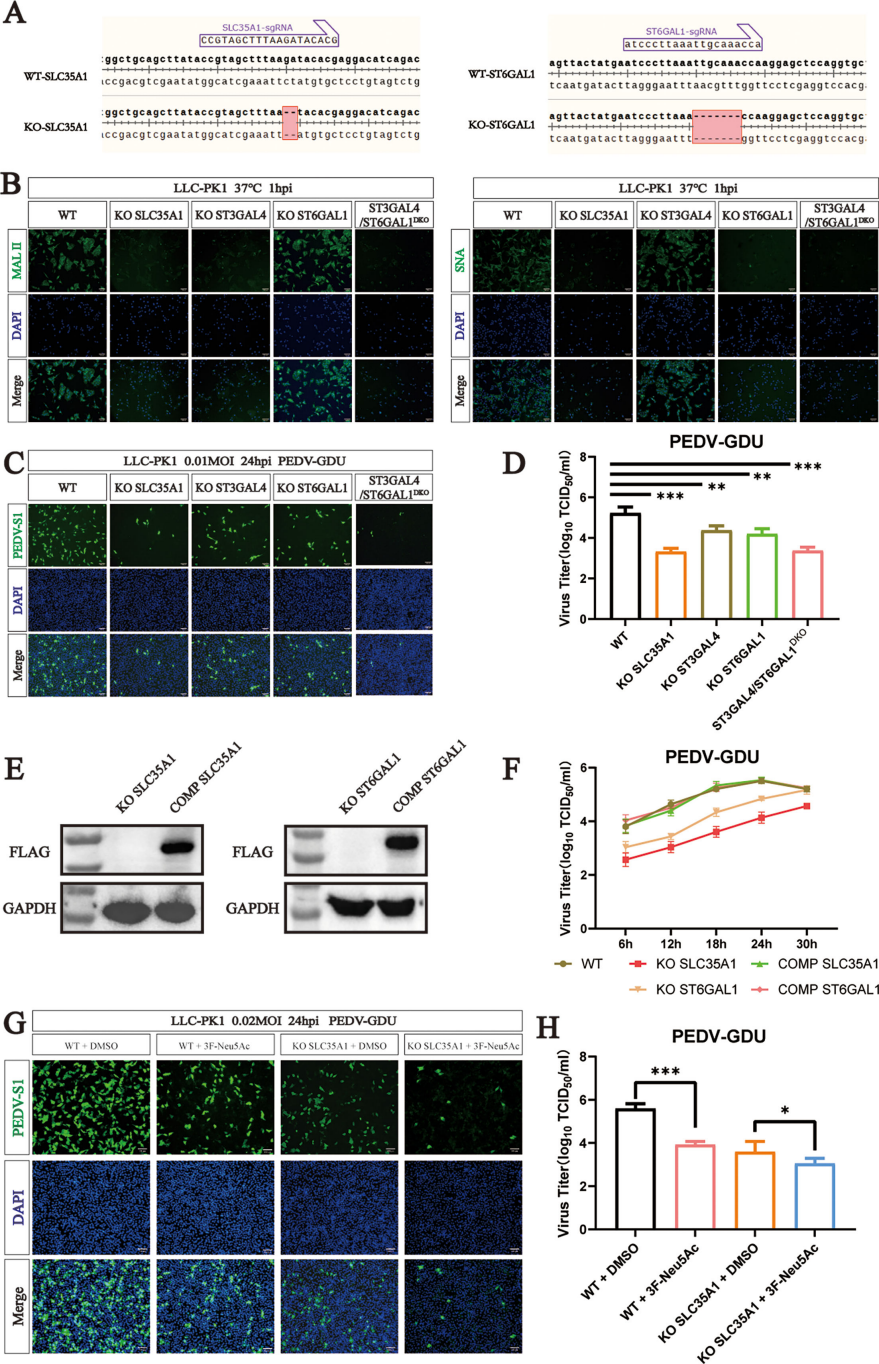
PEDV can use both α2,6-linked and α2,3-linked sialic acid to infect cells. (**A**) Alignment of the nucleic acid sequences of WT, SLC35A1, and ST6GAL1 knockout (KO) cells. (**B**) MAL II (binds to Sia-α2,3-galactose-GlcNAc) staining of WT and KO cells. SNA (binds to Sia-α2,6-galactose-GlcNAc) staining of WT and KO cells. Scale bar, 100 µm. (**C**) Immunofluorescence assays for detection of the PEDV in WT and KO cells following infection with PEDV (MOI = 0.1) at 24 hpi. Scale bar, 100 µm. (**D**) PEDV titers in WT and KO cells infected with PEDV (MOI = 0.1) at 24 hpi were evaluated by TCID_50_ assay. (**E**) Western blot analysis to detect the expression of SLC35A1-Flag in SLC35A1-KO and KO-SLC35A1 complement LLC-PK1 cells. Western blot analysis to detect the expression of ST6GAL1-Flag in ST6GAL1-KO and KO-ST6GAL1 complement LLC-PK1 cells. (**F**) One-step growth curves of WT, SLC35A1 KO, KO-SLC35A1 complement, ST6GAL1 KO, and KO-ST6GAL1 complement LLC-PK1 cells infected with PEDV (MOI = 0.1) measured by TCID_50_ assay. (**G**) Immunofluorescence assays for detection of the PEDV-S1 protein in cells following infection with PEDV (MOI = 0.2) at 24 hpi. Scale bar, 100 µm. (**H**) PEDV titers in cells infected with PEDV (MOI = 0.2) at 24 hpi were evaluated by TCID_50_ assay. *P* values were determined by two-tailed unpaired *t*-tests. Error bars represent standard deviations from three independent experimental replicates. ns, no significant; *P < 0.05; **P < 0.01; ***P < 0.001; and ****P < 0.0001. Data are representative of at least three independent experiments.

Among the sialyltransferase proteins, ST3GAL4 catalyzes the transfer of sialic acid to sugar chains through α2,3-linked linkages. To assess which subtypes of sialic acid have high affinity for PEDV infection, we constructed KO cells of sialic acid synthesis-related genes (SLC35A1 and ST6GAL1) and ST3GAL4/ST6GAL1 double KO LLC-PK1 cells (ST3GAL4/ST6GAL1^DKO^) (Fig. 3A). *Maackia amurensis* lectin II (MAL II) specifically binds to α2,3-linked sialic acid, and *Sambucus nigra* lectin (SNA) preferentially binds α2,6-linked sialic acid. Lectin binding analysis revealed a significant decrease in the synthesis of α2,3-linked sialic acid between SLC35A1, ST3GAL4, and ST3GAL4/ST6GAL1^DKO^ cells, and KO SLC35A1, ST6GAL1, and ST3GAL4/ST6GAL1^DKO^ had a strong effect on the synthesis of α2,6-linked sialic acid (Fig. 3B). To evaluate PEDV infection, we performed immunofluorescence assays targeting the PEDV-S1 and TCID_50_ assays for both WT and KO cells. Compared to control cells, all KO cell lines (ST3GAL4, SLC31A1, ST6GAL1, and ST3GAL4/ST6GAL1^DKO^) showed significantly reduced PEDV infection. Notably, the SLC35A1 KO and ST3GAL4/ST6GAL1^DKO^ cells demonstrated the strongest inhibitory effects (Fig. 3C and D).

To further examine the role of α2,6-linked sialic acid, we constructed SLC35A1 and ST6GAL1 complementation cell lines, which stably expressed the SLC35A1-Flag and ST6GAL1-Flag gene (Fig. S1B and C). Western blot analysis using the Flag-tag antibody confirmed successful expression (Fig. 3E). CCK-8 kit assay determined that neither KO nor complementation of SLC35A1 or ST6GAL1 significantly affected cell viability (Fig. S2A and B). Consistent with results from ST3GAL4 complementation, both SLC35A1 and ST6GAL1 complementation restored sensitivity to PEDV infection (Fig. 3F).

To assess whether combined inhibition enhances the antiviral effect, WT cells were pretreated with 3Fax-Neu5Ac, a specific sialyltransferase inhibitor. Cell viability was unaffected by treatment with 100 µM 3Fax-Neu5Ac (Fig. S3A). Compared to the control group, pretreatment with 3Fax-Neu5Ac significantly reduced PEDV replication in a dose-dependent manner (Fig. S3B). Furthermore, WT and KO-SLC35A1 cells were pretreated with 3FAx-Neu5Ac. In WT cells, 100 µM 3FAx-Neu5Ac significantly inhibited the PEDV replication compared to DMSO controls. Interestingly, PEDV replication was further decreased in KO-SLC35A1 cells pretreated by 3FAx-Neu5Ac compared to DMSO-treated KO-SLC35A1 cells, suggesting an enhanced antiviral effect from the combination of genetic KO and chemical inhibition (Fig. 3G and H). Collectively, these results indicated that PEDV relies on both α2,6-linked and α2,3-linked sialic acid for efficient cell entry and infection.

### Sialic acid facilitates PEDV attachment and internalization

**Fig 4:**
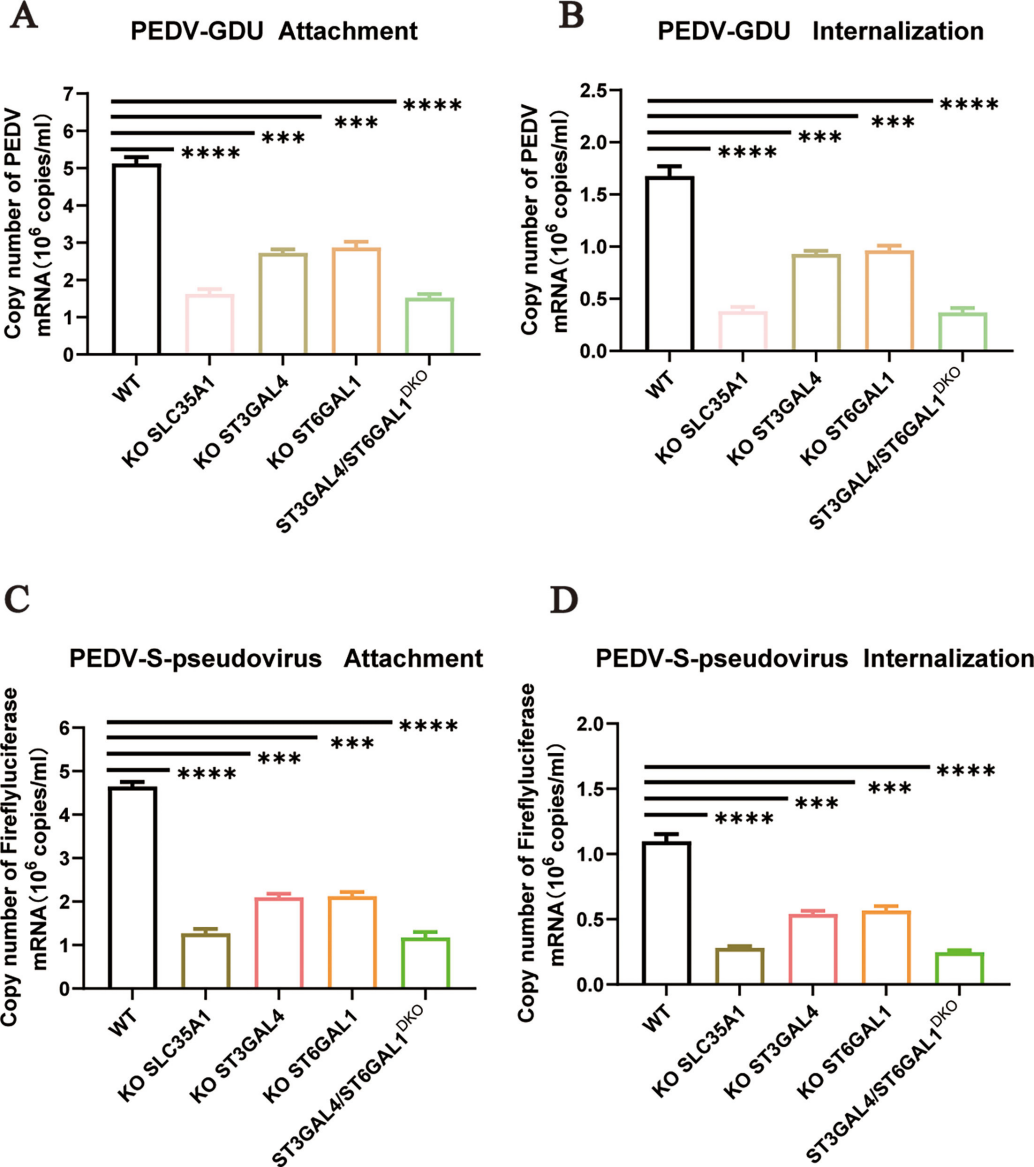
Sialic acid facilitates PEDV attachment and internalization. (**A**) The adsorption of PEDV on the WT, ST3GAL4 KO, and KO-ST3GAL4 complement LLC-PK1 cells, and the ST6GAL1 KO and KO-ST6GAL1 complement cells were evaluated by qRT‒PCR. The cells were infected with PEDV-GDU (MOI = 10) at 4°C for 1 h to evaluate the adsorption of PEDV. (**B**) The internalization of PEDV on the WT, ST3GAL4 KO, and KO-ST3GAL4 complement, and ST6GAL1 KO and KO-ST6GAL1 complement LLC-PK1 cells were evaluated by qRT‒PCR. The cells were incubated with PEDV (MOI = 10) at 4°C for 1 h, transferred to 37°C for 30 min and washed two times with acidic PBS (pH = 1.3) at 4°C to remove noninternalized virus particles. (**C**) The adsorption of PEDV on the WT, ST3GAL4 KO, and KO-ST3GAL4 complement LLC-PK1 cells, and the ST6GAL1 KO and KO-ST6GAL1 complement cells were evaluated by qRT‒PCR. The cells were infected with PEDV-S-pseudovirus (MOI = 10) at 4°C for 1 h to evaluate the adsorption of PEDV. (**D**) The internalization of PEDV on the WT, ST3GAL4 KO, KO-ST3GAL4 complement, and ST6GAL1 KO and KO-ST6GAL1 complement LLC-PK1 cells were evaluated by qRT‒PCR. The cells were incubated with PEDV-S-pseudovirus (MOI = 10) at 4°C for 1 h, transferred to 37°C for 30 min and washed two times with acidic PBS (pH = 1.3) at 4°C to remove noninternalized virus particles. *P* values were determined by two-tailed unpaired *t*-tests. Error bars represent standard deviations from three independent experimental replicates. ns, no significant; *P < 0.05; **P < 0.01; ***P < 0.001; and ****P < 0.0001. Data are representative of at least three independent experiments.

Our studies confirmed the importance of sialic acid in PEDV infection. To evaluate its role in the viral infection cycle, we performed virus attachment and internalization assays. LLC-PK1 (WT), KO-SLC35A1, KO-ST3GAL4, KO-ST6GAL1, and ST3GAL4/ST6GAL1^DKO^ cells were incubated with PEDV for 1 h at 4°C to allow virus absorption. The attachment efficiency was detected by real-time fluorescence quantitative PCR (qRT‒PCR). Compared to WT cells, KO cell lines showed a significant reduction in virus attachment (Fig. 4A). In the internalization assay, the cells were washed three times with acidic PBS (pH = 1.3) at 4°C to remove noninternalized virus particles. The assay showed that viral internalization was also reduced in cells lacking sialic acid synthesis-related genes (Fig. 4B). These findings were corroborated using PEDV-S-pseudovirus. KO of α2,3-linked or α2,6-linked sialic acid synthesis in cells also impaired PEDV-S-pseudovirus attachment and internalization (Fig. 4C and D).

### PEDV spike protein preferably binds α2,3-linked and α2,8-linked sialic acid

**Fig 5:**
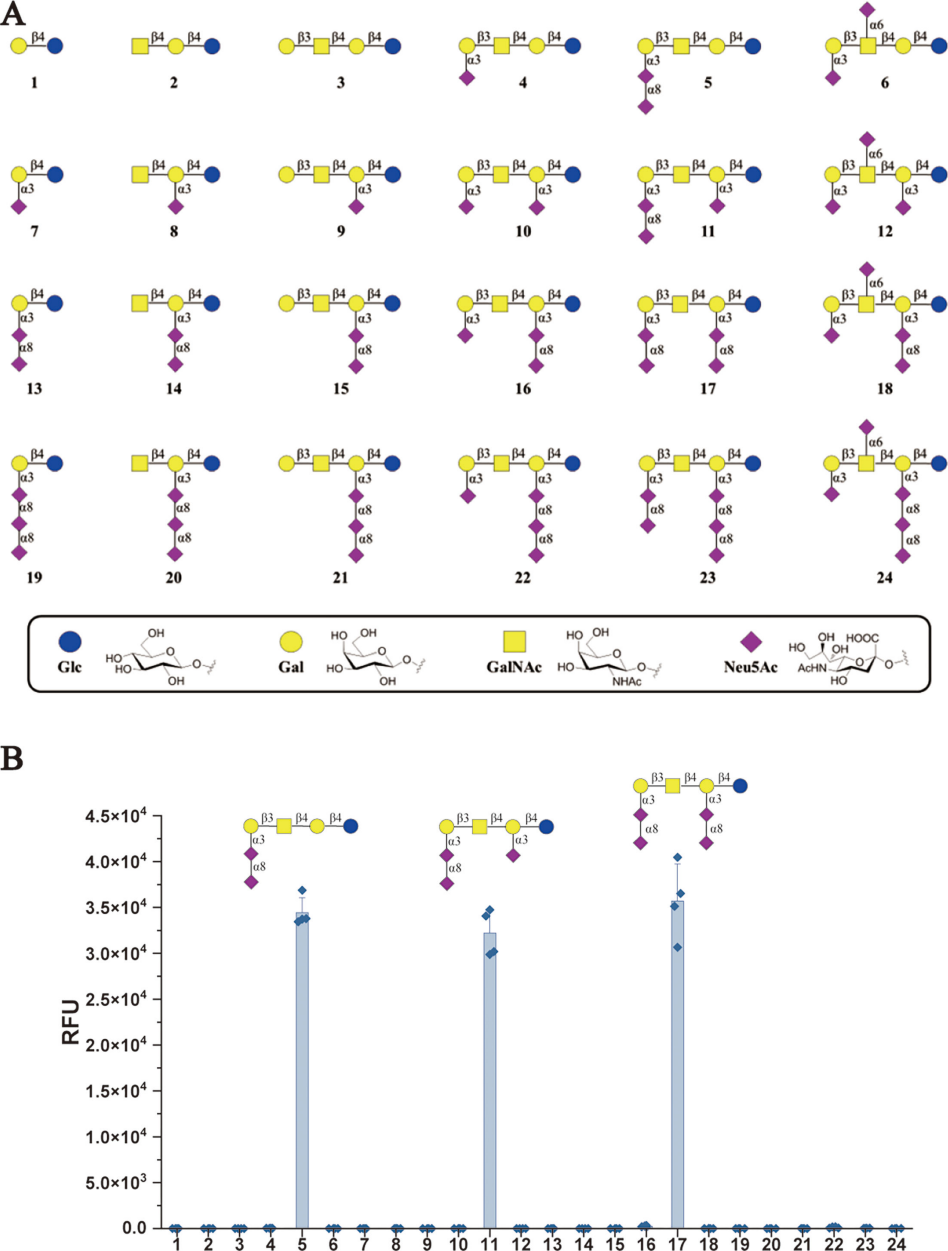
PEDV spike protein preferably binds α2,3-linked and α2,8-linked sialic acid. (**A**) The synthetic glycans 1–24 were used for microarray screening of the binding with PEDV S1. (**B**) Glycan microarray screen to identify which type of sialic acid for PEDV S1 binds. The average relative fluorescence value (RFU) was calculated by four independent replicates on the glycan microarray after the removal of the highest and lowest signals. The error bars indicate the standard deviation (SD) of RFU.

To assess the sialoglycan specificity of PEDV, we performed a glycan microarray screening using the PEDV S1 protein. Among all types of glycans screened, we find that α2,3-linked and α2,8-linked sialosides were most favored by PEDV S1. In particular, the S1 protein preferred binding to sialylated glycans containing terminal α2,8-Neu5Ac-α2,3-Neu5Ac epitope with or without internal α2,3-Neu5Ac or α2,8-Neu5Ac-α2,3-Neu5Ac motifs (Fig. 5). However, terminal α2,3-linked sialosides exhibited relatively weaker or negligible binding in the glycan array, contrasting with our cell-based assays where they serve as key attachment factors for PEDV. This discrepancy may be due to the ability of low-affinity glycans to engage in multivalent interactions on the cell surface, thereby enhancing virus binding and leading to biologically relevant interactions not captured in the array format. Notably, unlike other forms of sialylation, α2,8-linkage is more protein specific and only found on a few glycoproteins in mammalian intestinal cells (30, 31). Hence, α2,3-linked sialic acid serves as preferred attachment factors for PEDV *in vivo*.

### α2,3-linked and α2,6-linked sialic acids are important for infection of porcine coronaviruses

**Fig 6:**
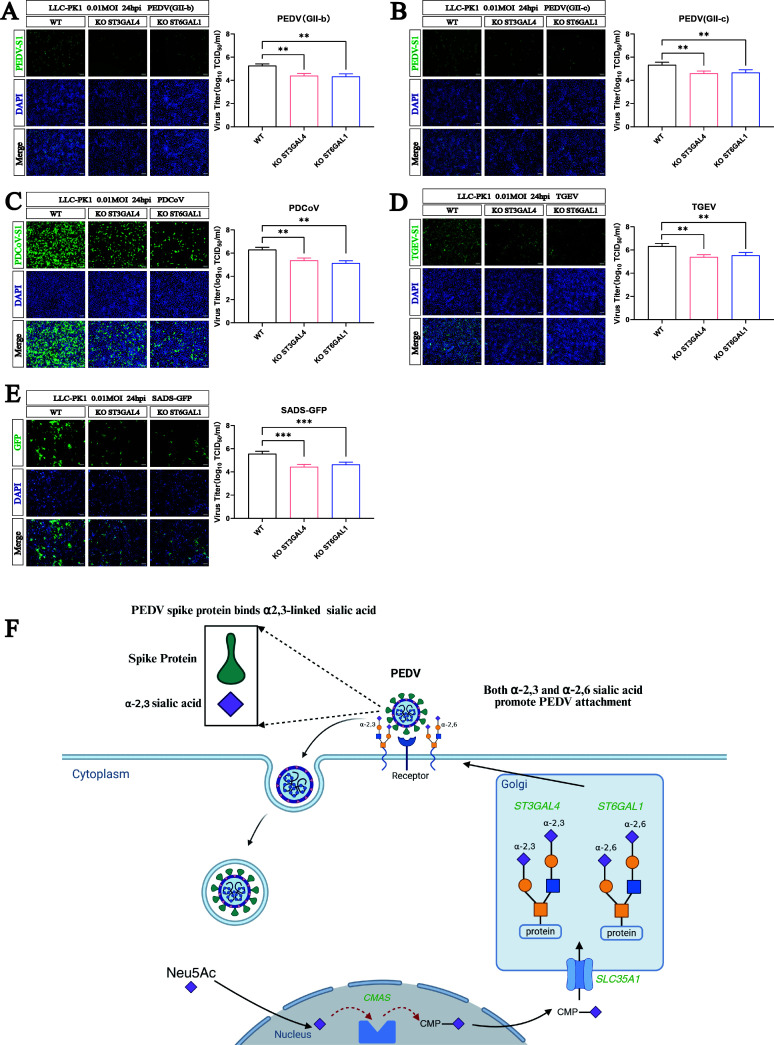
α2,3-linked and α2,6-linked sialic acids are essential for multiple coronaviruses infection. (**A**) Immunofluorescence assays (IFAs) for detection of the PEDV-S1 protein in WT and KO LLC-PK1 cells infected with PEDV(GII-b) (MOI = 0.1 at 24 hpi). Scale bar, 100 µm. Viral titers were measured by TCID_50_ assay. (**B**) IFAs for detection of the PEDV-S1 protein in WT and KO LLC-PK1 cells infected with PEDV(GII-c) (MOI = 0.1 at 24 hpi). Scale bar, 100 µm. Viral titers were measured by TCID_50_ assay. (**C**) IFAs for detection of the PDCoV-S1 protein in WT and KO LLC-PK1 cells infected with PDCoV (MOI = 0.1 at 24 hpi). Scale bar, 100 µm. Viral titers were measured by TCID_50_ assay. (**D**) IFAs for detection of the TGEV-S1 protein in WT and KO LLC-PK1 cells infected with TGEV (MOI = 0.1 at 24 hpi). Scale bar, 100 µm. Viral titers were measured by TCID_50_ assay. (**E**) IFAs for detection of the GFP protein in WT and KO LLC-PK1 cells infected with SADS-GFP (MOI = 0.1 at 24 hpi). Scale bar, 100 µm. Viral titers were measured by TCID_50_ assay. (F) Proposed model illustrating the different steps in synthesis of Sia and sialylated glycans. PEDV can use both α2,6-linked and α2,3-linked sialic acid to infect cells. *P* values were determined by two-tailed unpaired *t*-tests. Error bars represent standard deviations from three independent experimental replicates. ns, no significant; *P < 0.05; **P < 0.01; ***P < 0.001; and ****P < 0.0001. Data are representative of at least three independent experiments.

Sialic acid has been shown to enhance the infection of a variety of viruses, leading us to speculate that sialic acid may also play a significant role in other porcine enteric CoVs. Therefore, we investigated the necessity of sialic acid for infection of major epidemic PEDV strains (genotype GII-b and GII-c) as well as PDCoV, transmissible gastroenteritis virus (TGEV), and swine acute diarrhea syndrome coronavirus (SADS-CoV). Immunofluorescence assay (IFA) and viral titration were performed to detect CoV infection in both WT cells and KO cells. Compared to WT cells, infection of these viruses was significantly reduced in cells lacking ST3GAL4 and ST6GAL1 (Fig. 6). These results demonstrate that sialic acid, particularly in α2,3-linked and α2,6-linked forms, is essential for the efficient infection of cells by multiple porcine enteric coronaviruses.

## DISCUSSION

PEDV is a highly contagious swine virus that has caused significant economic damage to the pig industry since the emergence of more virulent strains in 2013. In this study, we conducted a genome-wide CRISPR/Cas9 screen to identify essential host factors that contribute to infection of such a highly virulent PEDV strain. Results from the screen and subsequent analysis highlighted that several genes involved in sialic acid synthesis, including ST3GAL4, ST6GAL1, and SLC35A1, are important for viral infection.

Sialic acids play a significant role in the infection process of various coronaviruses, including PEDV (11, 13). Modifications as well as glycan linkage of sialic acids can determine specificity of virus binding. Human IAVs have a greater affinity for α2,6-linked sialic acid (32), while avian IAVs are more inclined to use α2,3-linked sialic acid (33). Swine IAVs have a high affinity for both α2,3-linked and α2,6-linked sialic acid (24). We here demonstrate that PEDV can use both α2,3-linked and α2,6-linked sialic acids as receptors for infection. Through gene KO experiments, we observed that reducing sialic acid levels on the cell surface strongly suppressed PEDV infection. Interestingly, while both α2,3-linked and α2,6-linked sialic acids are implicated in PEDV infection, the double KO of ST3GAL4 and ST6GAL1, as well as the depletion of SLC35A1, which impacts both types of sialic acid, had the most substantial effect on virus binding, internalization, and infection, suggesting that PEDV may exploit multiple sialic acid subtypes to facilitate infection.

Interestingly, we found that the PEDV replication was decreased in SLC35A1 KO cells pretreated with 3Fax-Neu5Ac compared to those pretreated with DMSO. This suggests that additional mechanisms may exist to reduce PEDV infection in a sialic acid-independent manner. Wang et al. reported that KO of SLC35A1 led to abnormal expression of multiple glycoproteins, including downregulation of pAPN and ACE2 (34), indicating that SLC35A1 KO may exert an antiviral effect beyond those mediated by sialic acid. Although KO of sialic acid synthesis-related genes significantly suppressed infection, it did not completely block virus entry. This suggests that sialic acids function as auxiliary receptors rather than primary receptors, similar to what has been observed for other coronaviruses including MERS-CoV and TGEV (35, 36). Binding to sialoglycan binding may promote virus binding to the cell surface either directly or indirectly. Structural studies on OC43 have shown that sialoglycan binding may trigger opening of the spike protein which enables binding to a proteinaceous receptor (37). The structural implications of sialic acid binding on the PEDV spike protein dynamics remain to be explored.

Glycan microarray screening showed that PEDV S1 preferentially binds to α2,3-linked and α2,8-linked sialic acids, rather than the α2,6 type. However, we also found that both α2,3-linked and α2,6-linked sialic acids are implicated in the entry of PEDV and PEDV-S pseudovirus. We propose that low-affinity sialic acids can efficiently enhance PEDV binding and entry. Supporting this, Liu et al. showed that low-affinity α2,6-linked sialic acids facilitate avian IAV binding and entry by hetero-multivalent interactions (38). The role of low-affinity sialic acid types in PEDV entry needs to be further explored.

The use of sialoglycans for PEDV infection may be strain dependent and is particularly associated with virulent strains of the non-S-Indel type (39). We found that two recent PEDV strains, GII-b and GII-c, prevalent in China from 2020 to 2023, can utilize both types of sialic acids, similar to the PEDV-GDU strain.

In addition, our findings, along with previous studies (27, 40, 41), show that sialic acids are involved in the infection of other porcine coronaviruses. Targeting sialic acid biosynthesis pathways may inform future efforts aimed at developing antiviral strategies.

## MATERIALS AND METHODS

### Cells and virus

Human embryonic kidney 293 cells stably expressing the SV40 large T antigen (HEK-293T), African green monkey kidney epithelial cells (Vero), Porcine kidney proximal tubular epithelial cells (LLC-PK1), and swine testicular (ST) cells were purchased from ATCC. All cells were maintained in Dulbecco’s modified Eagle medium (Gibco, Shanghai, China) supplemented with 10% heat-inactivated fetal bovine serum and 1% penicillin/streptomycin (Cat#15140122, Gibco, Shanghai, China) were incubated at 37°C with 5% CO_2_.

The following viruses were used: the virulent PEDV strain GDU (GenBank accession number KU985230), the PDCoV strain (GenBank accession number MF095123.1), the TGEV strain WH-1 (GenBank accession number HQ462571.1), and SADS-GFP was kindly provided by Dr. Yao-Wei Huang (42). PEDV strain (GII-b: PP472643.1 and GII-c: PP472642.1) were isolated from our laboratory. The PEDV and SADS-GFP strain was propagated in Vero cells in the presence of 10 µg/mL trypsin, and the PDCoV strain was propagated in LLC-PK1 cells in the presence of 5 µg/mL trypsin.

### Reagents and antibodies

Mouse anti-PEDV-S1, Flag tag, PDCoV-S1, and TGEV-S1 monoclonal antibodies were prepared in our laboratory. Antibodies and dyes were purchased, including Alexa Fluor 488 Donkey anti-Mouse IgG (antGene, Wuhan, China), DAPI and Hoechst (Beyotime, Shanghai, China), FITC-labeled SNA and FITC-labeled MAL II lectins (Vector Lab, Burlingame, USA).

### Genome-wide CRISPR/Cas9 library screen

Genome-wide sgRNA library was generated by ±260,000 unique sgRNA in Huh7-Cas9 cells as described before (43). The coverage of genome-wide sgRNA library was >400-fold (44). For the genome-wide CRISPR screening assays, ~100 million Huh7-Cas9 library cells were infected with PEDV at an MOI of 0.1. At 21 days after infection, the surviving cells were expanded for NGS analysis and the next round of infection.

### Lentivirus preparation

Lentiviral vector, pMD2.G,, and psPAX2 plasmid were co-transfected in HEK 293T cells via jetPRIME transfection reagent (Polyplus, Paris, France) according to the manufacturer’s instructions. After 48 h postinfection (hpi) of transfection, the supernatant was collected and stored at −80°C.

### Generation of KO cell lines

All the sgRNAs used in this experiment were designed according to the dedicated website (http://crispor.tefor.net/crispor.py). The sgRNA sequence targeting the target genes was cloned and inserted into the LentiGuide-Puro vector to produce the recombinant lentivirus. LLC-PK1-Cas9 cells were infected with LentiGuide-Puro lentivirus, and puromycin (2.5 µg/mL) was added to select the positive cells at 24 hpi. We used the serial dilution method to select monoclonal cells. All primers used are listed in Table S1.

### Cell viability assay

A colorimetric-based cell counting kit-8 (CCK-8) assay (Dojindo Molecular Technologies, Rockville, MD, USA) was used to determine cell viability. In brief, control and KO cells were seeded in 96-well plates, and then 10 µL of CCK-8 reagent was added to each well at 12, 24, and 36 h. The plates were incubated in a 37°C constant temperature incubator for 4 h, and the absorbance at 450 nm was measured with a microplate reader.

### TCID_50_ and plaque assays

The stock virus was serially diluted to inoculate confluent Vero cell monolayers and grown in 96-well plates. The cell monolayer was washed three times with PBS before infection with 100 µL of the virus. Eight replicate wells were inoculated for each dilution, and the plates were incubated at 37°C with 5% CO_2_ for 2 days. The viral titers were calculated by using the Reed-Muench method and are expressed as TCID_50_ per milliliter.

### IFA

The cells were gently washed in PBS and then fixed with 4% paraformaldehyde for 10 min at room temperature. This was followed by a permeabilization step using ice-cold 0.1% Triton X-100 was added for 15 min at room temperature. Then, the samples were washed three times with PBS, and the cells were then blocked with 3% bovine serum albumin (BSA) in PBS for 30 min at room temperature. Afterward, the cells were incubated with an anti-PEDV spike antibody in PBS for 1 hpi at 37°C, followed by three washes with PBS. Finally, the cells were incubated with Alexa 488-labeled anti-human antibody for 45 min at 37°C, washed in PBS, and treated with DAPI at room temperature for 5 min to stain the nuclei.

### Western blot assay

Cells were lysed by RIPA and NP40 lysis buffer (Beyotime, Shanghai, China) containing 1 mM phenylmethanesulfonyl fluoride (Beyotime, Shanghai, China), then denaturation at 95°C for 5 min. The protein samples were resolved by 10% sodium dodecyl sulfate-polyacrylamide gel electrophoresis and transferred to polyvinylidene difluoride membranes (Millipore, USA) that were blocked with PBS containing 5% skim milk at room temperature for 2 h. Membranes were incubated in TBST containing primary antibodies overnight at 4°C. On the following day, membranes were washed and incubated with horseradish peroxidase-conjugated anti-mouse secondary antibodies for 1 h at room temperature. Immunoreactive bands were visualized using the ChemiDoc MP Imaging System (Bio-Rad, USA).

### Absolute quantitative real-time PCR analysis

Viral RNA from cell suspensions was extracted using the Viral RNA Extraction kit (TaKaRa Bio, Japan) following the manufacturer’s instructions. The cDNA was obtained by RT‒PCR using HiScript III 1st Strand cDNA Synthesis Kit (Vazyme #R312, China). The absolute qRT‒PCR assay for quantifying the PEDV genome was performed with RealUniversal SYBR Green Premix (Tiangen, Beijing, China) following the manufacturer’s instructions. Following the program: one cycle of 30 s at 95°C, followed by 45 cycles of 5 s at 95°C and 30 s at 60°C. PEDV N protein coding sequence was cloned and inserted into the pMD19-T vector as a standard reference for the quantification of PEDV copy numbers. Table S1 lists primers utilized in qRT‒PCR (45).

### Lectin assays

The cells were washed in PBS, then fixed with 4% formaldehyde for 10 min, washed with PBS two times and incubated with 20 µg/mL FITC-labeled lectins for 1 hpi on ice, washed in PBS and treated with Hoechst at room temperature for 5 min to stain the nuclei. A microscope (EVOS FL, Thermo Fisher Scientific, USA) was used to obtain images.

### Virus attachment and internalization assay

Virus attachment and internalization were assayed by qRT‒PCR analysis. For the attachment assay, plated cells were washed three times with ice-cold PBS, incubated with PEDV-GDU (MOI = 10) at 4°C for 1 hpi, and the cells were washed with ice-cold PBS to remove unbound virus. For the internalization assay, plated cells were washed three times with ice-cold PBS, incubated with PEDV-GDU (MOI = 10) at 4°C for 1 hpi, and then supernatant was removed. The cells were transferred to 37 ℃ for 30 min that allowed virus entry, then washed three times with acidic PBS (pH = 1.3) at 4°C to remove noninternalized particles.

### Protein expression

The ST-tagged pA-LS protein was expressed in HEK293F cells. Briefly, the plasmids were transfected using polyethylenimine reagent and the medium was replaced with 293 SFM II-based expression medium (Gibco Life Technologies) 6 h posttransfection. After 5–6 days, the culture supernatants were collected and the expressed proteins were purified via StrepTactin Sepharose beads (IBA) according to the manufacturer’s instruction. The Fc-tagged PEDV-S1 protein was expressed in GnTI (N-acetylglucosaminyltransferase I)-KO HEK-293T cell lines, which were generated as described previously (46). The fusion proteins were purified using pA affinity chromatography (17-0780-01; GE Healthcare) following the manufacturer’s instructions.

### Glycan microarray printing and screening

The synthetic ganglioside glycans 1–24 (100 µM) were dissolved in sodium phosphate buffer (pH 8.5, 250 mM) and printed in replicates of six with spot volume ~400 pl at 55% humidity (47). The slides were incubated overnight in a saturated NaCl chamber (an incubation chamber with 75% relative humidity, maintained by saturated NaCl-soaked cotton), followed by quenching with 50 mM ethanolamine in a Tris buffer (pH 9.0, 100 mM). The blocked slides were dried by centrifugation and kept in a desiccator at room temperature prior to use. Microarray slides were blocked with TSM blocking buffer (20 mM Tris Cl, pH 7.4, 150 mM NaCl, 2 mM CaCl_2_, 2 mM MgCl_2_, 0.1% Tween-20, and 5% skimmed milk). Purified pA-LS and PEDV-S1-Fc were uniformly mixed according to a molar ratio of 1:1 and incubated for 30 min on ice, and then empty pA-LS nanoparticles or nanoparticles complexed with PEDV-S1-Fc were incubated for 120 min at 4°C in TSM binding buffer (20 mM Tris Cl, pH 7.4, 150 mM NaCl, 2 mM CaCl_2_, 2 mM MgCl_2_, 0.05% Tween, and 1% BSA) on the microarray slides. After two repeated washes with TSM washing buffer (20 mM Tris-Cl, 150 mM NaCl, 2 mM CaCl_2_, 2 mM MgCl_2_, and 0.05% Tween) and TSM buffer (20 mM Tris-Cl, pH 7.4, 150 mM NaCl, 2 mM CaCl_2_, and 2 mM MgCl_2_) respectively, the subarrays were incubated with 100 µL StrepMAB-Classic DY-649 (diluted 1:200 in TSM binding buffer) and washed with TSM washing buffer, TSM buffer, and deionized water. The resulting array slides were scanned for fluorescence on an InnoScan 710 Microarray Scanner. The data were processed with Mapix software and further analyzed using IBM SPSS 19 (SPSS Inc.). Relative fluorescence values (RFUs) were calculated by averaging four independent replicates (corrected for mean background) on the glycan array after removal of the highest and lowest signals.

### Statistical analysis

All the statistical analyses, except for the genetic screening and single-cell sequencing data, were performed using GraphPad Prism software. Two-tailed unpaired *t*-tests were used to compare two unpaired groups. The data were analyzed for normality and log (normal distribution) using the Kolmogorov–Smirnov test before analysis of variance was applied. All analyses were performed at a threshold *α* level of 0.05. The specific statistical tests used for each data set are mentioned in the respective figure captions, as well as any data transformation that was applied. All measurements were taken from distinct samples.

## SUPPLEMENTAL MATERIAL

Supplemental Material mbio.01628-25-s0001.pdf Figures S1 to S3; Table S1.

ASM does not own the copyrights to Supplemental Material that may be linked to, or accessed through, an article. The authors have granted ASM a non-exclusive, world-wide license to publish the Supplemental Material files. Please contact the corresponding author directly for reuse.
