# Supplementary material for: Genome-wide CRISPR screen reveals key role of sialic acids in PEDV and porcine coronavirus infections
Source: mBio. 2025 Aug 6;16(9):e01628-25. doi: 10.1128/mbio.01628-25 (PMC12421841; doi:10.1128/mbio.01628-25)
Supplement: Supplemental Material — Figures S1 to S3; Table S1. [file mbio.01628-25-s0001.pdf]

Supplementary Information

Genome-wide CRISPR screen reveals key role of sialic acids in PEDV and porcine coronavirus infections

Guanghao Guo[1,#], Mengjia Zhang[1,#], Zhuojia Xu[2], Peng Xi[1], Hongmei Zhu[1], Anouk Evers[3], Robert Jan Lebbink[3], Yifei Lang[4], Qigai He[1], Yao-Wei Huang[5], Tiehai Li[2,*], Berend Jan Bosch[6,*], Wentao Li[1,*]

Supplementary Figure 1

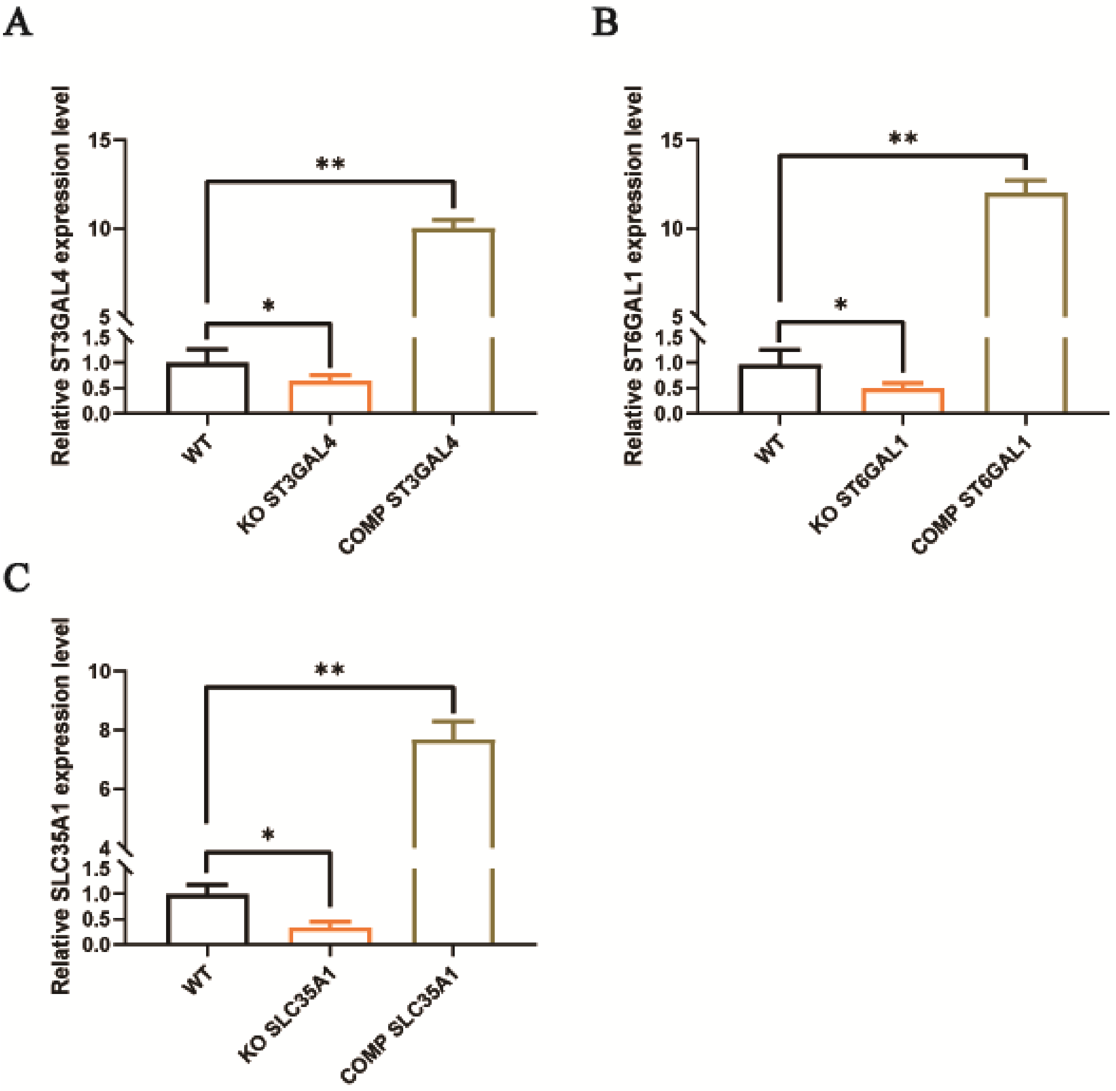

Supplementary Figure 1. RT-qPCR assay for determination of endogenous relative mRNA level. (A) RT-qPCR assay for determination of relative mRNA level of ST3GAL4. (B) RT-qPCR assay for determination of relative mRNA level of ST6GAL1. (C) RT-qPCR assay for determination of relative mRNA level of SLC35A1.

Supplementary Figure 2

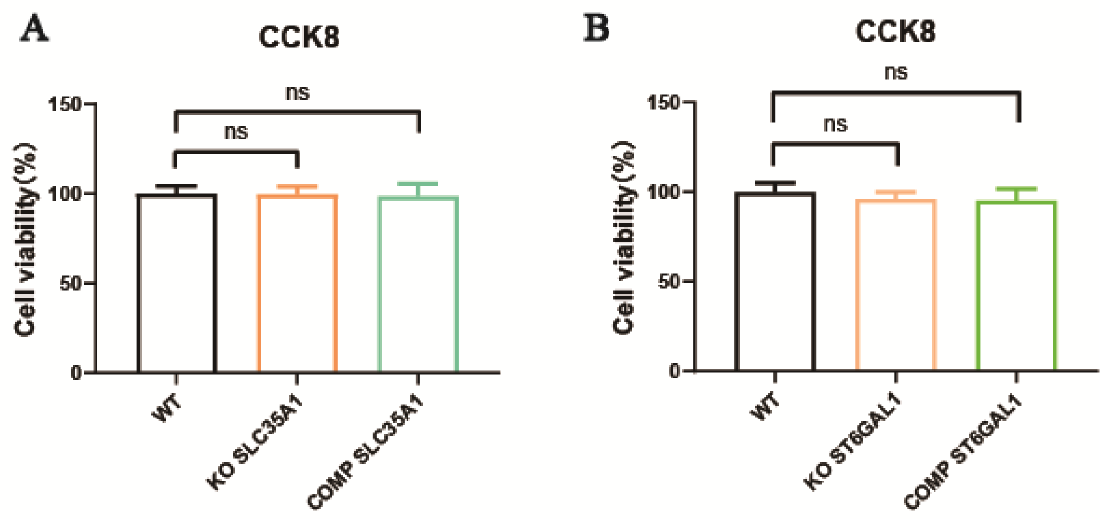

Supplementary Figure 2. CCK-8 assay determined WT, knockout and complementation cell viability (A) Cell viability detection in WT, SLC35A1 KO and KO-SLC35A1 complement LLC-PK1 cells by a CCK-8 kit. (B) Cell viability detection in WT, ST6GAL1 KO and KO-ST6GAL1 complement LLC-PK1 cells by a CCK-8 kit.

Supplementary Figure 3

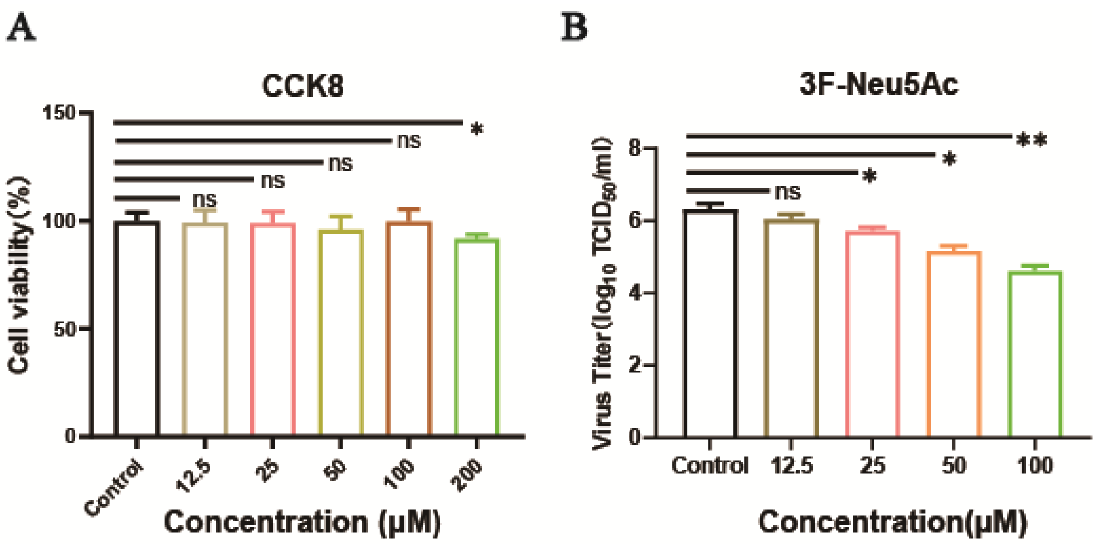

Supplementary Figure 3. Effects of 3F-Neu5Ac on PEDV infection. (A) LLC-PK1 cells were treated with or without the 3F-Neu5Ac for 12 hours, and cell viability was assessed by CCK-8 assay. (B) LLC-PK1 cells were pretreated with different concentrations of 849 the 3F-Neu5Ac (12.5, 25, 50, 100 μM) for 1 hour, followed by PEDV infection (MOI of 0.01). The PEDV titers were determined at 24 hpi. *P* values were determined by two-tailed unpaired *t*-tests. Error bars represent standard deviations from three independent experimental replicates. ns, no significant; $*p < 0.05$; $**p<0.01$; $***p<0.001$; $****p<0.0001$. Data are representative of at least three independent experiments.

Supplementary Table 1

**Table Supplementary 1. Primer pairs and sgRNAs targeting sequences used in this study**		
**Name**	**Sequence**	**Notes**
PEDV-N-qPCR-F	ATCGACCACATGGCTCCAA	for real-time quantitative PCR assay
PEDV-N-qPCR-R	CAGCTCTTGCCCATGTAGCTT	
ST3GAL4-qPCR-F	GGAGCAGAGACAAGAGAGGT	
ST3GAL4-qPCR-R	TCTCAAGTAGCAGGGTTCCT	
ST6GAL1-qPCR-F	GTGCTGAGGAGTCTGGAGAA	
ST6GAL1-qPCR-R	AGAGCTGTCCTTGTTCCACA	
SLC35A1-qPCR-F	TGAAGTTGAGTGTGCCTTCC	
SLC35A1-qPCR-R	AGCAGTGCAGGGAATCTTCA	
Luciferase-qPCR-F	GGACATTACCTACGCCGAGT	
Luciferase-qPCR-R	CTATTCTCGCTGCACACCAC	
DIPK2A-sgR-F	ttggAACGTCTACTTCGCGCAGTA	for construction of lentivirus sgRNA expression vectors
DIPK2A-sgR-R	aaacTACTGCGCGAAGTAGACGTT	
ST3GAL4-sgR-F	ttggCCGGGATGACAGCTCTCCCC	
ST3GAL4-sgR-R	aaacGGGGAGAGCTGTCATCCCGG	
FOXA2-sgR-F	ttggAAGGGCACGAGCCGTCCGAC	
FOXA2-sgR-R	aaacGTCGGACGGCTCGTGCCCTT	
PATZ-sgR-F	ttggGACGCTTCTTGCGGCCCGTC	
PATZ-sgR-R	aaacGACGGGCCGCAAGAAGCGTC	
SLC35A1-sgR-F	ttggCCGTAGCTTTAAGATACACG	
SLC35A1-sgR-R	aaacCGTGTATCTTAAAGCTACGG	
ST6GAL1-sgR-F	ttggATCCCTTAAATTGCAAACCA	
ST6GAL1-sgR-R	aaacTGGTTTGCAATTTAAGGGAT	
